# Treatment with Toll-like Receptor (TLR) Ligands 3 and 21 Prevents Fecal Contact Transmission of Low Pathogenic H9N2 Avian Influenza Virus (AIV) in Chickens

**DOI:** 10.3390/v15040977

**Published:** 2023-04-16

**Authors:** Sugandha Raj, Ayumi Matsuyama-Kato, Mohammadali Alizadeh, Nitish Boodhoo, Eva Nagy, Samira Mubareka, Khalil Karimi, Shahriar Behboudi, Shayan Sharif

**Affiliations:** 1 Department of Pathobiology, Ontario Veterinary College, University of Guelph, Guelph, ON N1G 2W1, Canada; 2 Sunnybrook Research Institute, Department of Laboratory Medicine and Pathobiology, University of Toronto, Toronto, ON M4N 3M5, Canada; 3 The Pirbright Institute, Pirbright, Woking GU24 0NE, Surrey, UK

**Keywords:** H9N2, AIV, transmission, fomites, chickens, feces, infection, spike, persistence, Toll-like-receptor, intestine, antiviral, interferons

## Abstract

Transmission of H9N2 avian influenza virus (AIV) can occur in poultry by direct or indirect contact with infected individuals, aerosols, large droplets and fomites. The current study investigated the potential of H9N2 AIV transmission in chickens via a fecal route. Transmission was monitored by exposing naïve chickens to fecal material from H9N2 AIV-infected chickens (model A) and experimentally spiked feces (model B). The control chickens received H9N2 AIV. Results revealed that H9N2 AIV could persist in feces for up to 60–84 h post-exposure (PE). The H9N2 AIV titers in feces were higher at a basic to neutral pH. A higher virus shedding was observed in the exposed chickens of model B compared to model A. We further addressed the efficacy of Toll-like receptor (TLR) ligands to limit transmission in the fecal model. Administration of CpG ODN 2007 or poly(I:C) alone or in combination led to an overall decrease in the virus shedding, with enhanced expression of type I and II interferons (IFNs) and interferon-stimulating genes (ISGs) in different segments of the small intestine. Overall, the study highlighted that the H9N2 AIV can survive in feces and transmit to healthy naïve chickens. Moreover, TLR ligands could be applied to transmission studies to enhance antiviral immunity and reduce H9N2 AIV shedding.

## 1. Introduction

Avian influenza viruses (AIV) are members of genus Influenza A viruses (AIV) within the family *Orthomyxoviridae* [1]. Influenza viruses are enveloped, single-stranded RNA viruses with a negative-sense segmented genome. Based on severity, AIV are categorized into high- and low-pathogenicity avian influenza viruses (HPAIV and LPAIV, respectively) [2]. Low-pathogenicity H9N2 AIV strains have been circulating in the Middle East, Central Asia, Africa and Europe [3]. These strains may cause mild to sub-clinical infections with a marked reduced meat and egg production and decreased body weight [4], posing significant economic losses to the poultry industry globally [3].

LPAIVs can infect a diverse species from avian to mammalian hosts, including waterfowl, domestic poultry, pigs, horses, whales and seals [3,5]. Transmission of H9N2 AIV in poultry has been widely recognized, and there have been recent reports of transmission of H9N2 AIV viruses to humans [6,7], which highlights the significance of the H9N2 subtype as a zoonotic pathogen.

AIV can persist in the biotic and abiotic components of the environment and spread by various transmission routes [8]. The direct route of transmission involves the transmission of AIV between infected and susceptible hosts that come in close/immediate contact to one another. The airborne transmission of AIV occurs by inhalation of fine particle aerosols (<5 μm) or large respiratory droplets >5–10 μm [9]. Indirect transmission can occur by exposure of birds to AIV-contaminated objects (fomites), feces of infected birds or via waterborne routes [10,11,12,13,14,15]. It has been previously demonstrated that H4N6, H5N1, H6N8, H3N6 and H3N2 AIV can survive in fecal material for different periods of time in the environment [16,17,18,19,20]. LPAIV can persist in poultry fecal droppings and remain infective for 24–48 h in wet manure and 48 h in litter material [12,15,21]. Additionally, Beard and colleagues (1984) showed persistence of H5N2 AIV in chicken feces for up to 7 days at 20 °C and 35–40 days at 4 °C [22]. However, what remains to be studied is whether AIV present in fecal matter can be a potential transmission source for naïve chickens.

H9N2 AIV can replicate within the upper respiratory and gastrointestinal tract (GIT) of chickens, and shedding can be detected using oral, tracheal and cloacal swabs from infected chickens [23,24,25]. Many studies have focused on exploring strategic ways to reduce H9N2 AIV replication, such as the administration of Toll-like receptor (TLR) ligands, vaccination, supplements and probiotics [25,26,27,28,29]. TLRs play a fundamental role in sensing the pathogens that invade host cells, which, in turn, may induce specific innate responses against the pathogen [30]. The induction of innate pro-inflammatory and anti-viral responses by TLR ligands in AIV infection has been widely reported in the spleen, lungs, cecal tonsils and mononuclear cells in chickens [29,31,32,33,34]. What remains to be studied is the efficacy of these TLR ligands in reducing H9N2 AIV transmission via different routes in transmission models. The current study was designed to establish a ‘fecal’ transmission model to determine whether H9N2 AIV can survive in feces and subsequently act as a source of transmission for exposed naïve chickens. The study further investigated the potential role of TLR ligands, including cytosine-phosphorothioate-guanine oligonucleotide (CpG ODN 2007) and polyinosinic:polycytidylic acid (poly(I:C)), when used alone or in combination, to minimize H9N2 AIV transmission. The study also investigated the underlying mechanisms through which TLR ligands can reduce H9N2 AIV transmission from infected to naïve chickens.

## 2. Materials and Methods

### 2.1. Chickens

One-day-old specific pathogen-free (SPF) White Leghorn chickens (*n* = 204) were purchased from the Canadian Food Inspection Agency (Ottawa, ON, Canada). The chickens were maintained in Horsfall units at the Research Isolation Unit at the University of Guelph. All experiments were approved by the Animal Care Committee (AUP 4203) at the University of Guelph and adhered to the guidelines of the Canadian Council on Animal Care.

### 2.2. Virus Propagation

An H9N2 LPAIV strain, A/TK/IT/13VIR1864-45/2013, was used for the present research. The virus strain was provided by Instituto Zooprofilattico Spermentale delle Venezie (IZSVe), Legnaro, Padua, Italy. To propagate the virus, 10-day-old embryonated chicken eggs were inoculated with H9N2 AIV and incubated for 72 h at 37 °C. Seventy-two hours post-incubation, the eggs were held overnight at 4 °C. The allantoic fluid was collected and centrifuged at 400× *g* for 15 min (mins) and stored at −80 °C. Virus quantification was done by titrating the virus on Madin–Darby canine kidney (MDCK) cells. The titers were calculated based on the endpoint dilutions expressed as 50% tissue culture infectious dose (TCID_50_/mL) [35].

### 2.3. Infectious Dose

Two independent transmission trials (models A and B) were performed. An inoculum containing 8 × 10^8^ TCID_50_ units of H9N2 AIV in 250 μL was used in both the models [24]. In model A, the seeder chickens were infected via a combination of the ocular, intra-tracheal and intra-nasal routes (50 μL/route).

To infect the exposed chickens in model B, i.e., the spiked fecal model, feces were collected from healthy/uninfected chickens (*n* = 10). The samples (*n* = 10) (negative for H9N2 AIV) were collected in 15 mL centrifuge tubes containing 10 mL of double distilled water (DDW), pH 7.0 (Life Technologies, Grand Island, NY, USA). The samples were spiked with 8 × 10^8^ TCID_50_ units of H9N2 AIV individually in each tube. The spiked fecal samples were then pooled, forming a final volume of 100 mL. The prepared inoculum was deposited in different locations of the Horsfall unit. 

### 2.4. TLR Ligands

Poly(I:C) was purchased from Sigma-Aldrich (Catalogue no. P9582, Oakville, ON, Canada) and the synthetic class B CpG ODN 2007 was obtained from Invivogen (San Diego, CA, USA). All ligands were re-suspended in phosphate buffer saline (PBS, pH 7.4) as per the manufacturer’s guidelines.

### 2.5. Experimental Design

The main objective of the present research was to determine whether AIV can transmit from H9N2 AIV-contaminated feces to naïve chickens. To address this, two independent transmission experiments (model A and B) were performed with chickens using Horsfall isolators. These isolators provide a constant temperature (90.5 °F) and humidity with minimal fluctuations throughout the trial period. The units were installed with non-absorbable/porous bedding material (TrafficMaster precut Artificial grass #BNC282115084-1, TrafficMaster, Vietnam) to facilitate maximum survival of H9N2 AIV and prevent any detrimental effect due to loss of moisture content in fecal material [36].

#### 2.5.1. Model A: Feces from H9N2 AIV-Infected Chickens

Trial 1 consisted of 14-day-old chickens (*n* = 40). The experimental setup comprised two sub-groups: a seeder group (*n* = 10) and an exposed group (healthy/uninfected) (*n* = 10). On day 14 of age, the seeder chickens were inoculated with H9N2 AIV through a direct inoculation method or with PBS in the negative control group (*n* = 10). The experimental setting within the unit, such as non-absorbable bedding mats, feeders and water fonts, was undisturbed during the first three days post-inoculation (PI). The seeder chickens were removed on day 3 PI from the isolators and replaced with naïve chickens (*n* = 10/ group). The seeder chickens were housed in a separate unit where they were swabbed at various time points to monitor virus shedding post-inoculation. The naïve chickens were exposed to feces from infected chickens for 14 days.

#### 2.5.2. Model B: Experimentally Spiked Feces

The second trial consisted of 14-day-old SPF chickens (*n* = 50). Fecal samples (*n* = 10) that tested negative for H9N2 AIV were collected from healthy/naïve chickens (*n* = 10) and spiked individually with H9N2 AIV. The prepared inoculum was deposited (poured) in different locations within the Horsfall unit, and a group of exposed chickens (*n* = 10/group) were then added to the H9N2 AIV-contaminated Horsfall unit. PBS was deposited in different locations within the isolator for the negative control. A direct contact transmission model was established as a positive control by infecting a seeder group (*n* = 10) and adding an exposed group (*n* = 10) of chickens 72 h PI. Both the seeder and exposed chickens were maintained for a period of 14 days post-exposure (PE). 

Another study used model B to determine the effects of CpG ODN 2007 and poly(I:C) on minimizing fecal contact transmission of H9N2 AIV. Fifty chickens were divided into five treatment groups (*n* = 10/group). Eighteen hours prior to the addition of the H9N2 AIV-spiked fecal inoculum to the isolator (except for the PBS+ unchallenged group), the exposed chickens were injected intramuscularly (i.m.) in the pectoral muscle with 100 μL of either CpG ODN 2007 (10 μg/chicken), poly(I:C) (400 μg/chicken) or a combination of CpG ODN 2007 (10 μg) + poly(I:C) (400 μg). The control groups received 100 μL PBS (PBS+ unchallenged and PBS+ challenged). The doses for the ligands used for the present study were determined from our previous studies [29,37]. 

Furthermore, to address the underlying mechanisms of the TLR ligands, the present study focused on determining the anti-viral responses in different parts of the small intestine (duodenum, jejunum and ileum). Sixty-four chickens were divided into four treatment groups (*n* = 18/group). On day 14 of age, the chickens were administered (i.m.) with 100 μL of either CpG ODN 2007 (10 μg/chicken), poly(I:C) (400 μg/chicken), a combination of CpG ODN 2007 (10 μg/chicken) + poly(I:C) (400 μg/chicken) or 100 μL PBS (PBS+ unchallenged) in the pectoral muscle. Chickens (*n* = 6) were euthanized at 3, 8 and 18 h after administration of TLR ligands. Tissues from the small intestines (2 cm of proximal, middle and distal portions of duodenum, jejunum and ilium) were collected and stored in RNAlater (Thermo Fisher Scientific Baltics UAB, Vilnius, Lithuania) at −80 °C until further processing.

### 2.6. Collection of Samples and Virus Titration

#### 2.6.1. Virus Isolation

To determine virus shedding in treatment groups, oral and cloacal swabs were collected from the seeder and exposed chickens on days 3, 5, 7 and 9 post-inoculation (PI) in the seeder groups and post-exposure (PE) in the exposed groups. Puritan PurFlock Ultra sterile flocked collection tubes (Gilford, ME, USA) were used for the collection of oral and cloacal swabs. The swab samples were transported on ice in 1.5 mL centrifuge tubes containing 1 mL of transport medium DMEM (Dulbecco’s Modified Eagle’s Medium) supplemented with 0.5% (bovine serum albumin) BSA fraction V, 10 mL penicillin (200 U/mL), streptomycin (80 μg/mL) and 5 mL gentamycin (50 μg/mL) to prevent any contamination. The swab samples were vortexed for 1 min and centrifuged at 500× *g* for 10 min at 4 °C. The supernatant from the swab samples was aliquoted and stored at −80 °C. 

Virus titers in swabs were quantified by serial dilution over MDCK monolayer cells and incubated at 37 °C for 72 h. The titers were based on detecting the highest endpoint dilution that shows a cytopathic effect (CPE) in the infected wells, confirmed by hemagglutination test with 0.5% chicken blood. Titers were expressed as TCID_50_/mL and calculated using the Reed–Muench formula [35]. 

#### 2.6.2. Virus Persistence

To test the persistence of H9N2 AIV in models A and B, fecal material (*n* = 10) was collected (after naïve birds were exposed) from various locations within the Horsfall isolators. For model A, fecal samples were collected on day 3 PI after addition of exposed chickens in the isolator (0 h PE). For model B, fecal samples were collected at different time points from the floor of the isolator immediately after depositing the spiked inoculum in the unit. The collected samples were placed in petri dishes within the Horsfall units. The petri dishes were tightly sealed with paraffin to prevent any fluctuations in temperature. To test the viability and infectivity of H9N2 AIV, samples were taken from the collected fecal material at specific time points. The samples were transported on ice in 5 mL centrifuge tubes containing 1.5 mL of transport medium. Fecal samples were processed by vortexing (1 min), followed by centrifugation at 500× *g* for 5 min to remove particulate matter. The clarified supernatant was aliquoted and stored at −80 °C until further use.

Infectious virus titers in the collected fecal samples were determined every 12 h from the point of addition of exposed chickens on day 3 PI (0 h PE) in model A. In model B, viability was determined every 12 h beginning from 0, 6 h after the addition of the exposed chickens (0 h PE), until no viable titers were detected in the fecal samples. The collected fecal samples were processed to quantify virus titers using the TCID_50_ assay. 

#### 2.6.3. pH

pH of the collected fecal samples (*n* = 10) was determined using pH indicator strips (colourpHAST^®^, Darmstadt, Hessen, Germany) of ranges 4.0–7.0 and 6.5–10 from the collected fecal samples of both models every 6 h PE along with the virus persistence. 

#### 2.6.4. Hemagglutination Inhibition (HI) Assay

Serum samples were used to determine the antibody titers on day 7 and 14 PE. A total of 50 μL of the serum samples was diluted (two-fold) in PBS. Fifty μL of H9N2 AIV containing 8 haemagglutinin units was added over the serum samples and incubated for 30 min at room temperature (RT) in 96-well V bottom plates (Corning Inc, Corning, NY, USA). A total of 0.5% of the chicken red blood cells (RBCs) were then added, and the plates were incubated for 30 min at RT. The HI titer was calculated as the reciprocal of the greatest dilution that demonstrated inhibition of red blood cell agglutination (log_2_ scale) [28].

#### 2.6.5. RNA Extraction, cDNA Synthesis and Real-Time PCR 

Total RNA extraction and cDNA synthesis was performed as described previously [32]. Real-time PCR was conducted using SyBR Green I Master Mix (Roche Diagnostics, Basel, Switzerland). The primer sequences (Table 1) used in the present study were synthesized by Sigma-Aldrich, Oakville, ON, Canada. The expression of the target genes was calculated relative to the housekeeping gene ß-actin using the LightCycler^®^ 480 II instrument (Roche Diagnostics, Basel, Switzerland) [32].

### 2.7. Statistical Analysis

A Pearson’s correlation test was used to observe the correlation between pH and virus load. In the second part of the study, gene expression and virus shedding results between multiple TLR ligand-treated groups were analyzed using Levene’s test to determine equality of variances, followed by one-way ANOVA and Tukey’s post hoc test. When the data did not have equal variances, the Kruskal–Wallis test was used for analysis. Gene expression analysis was done relative to the housekeeping gene β-actin and compared to the PBS+ unchallenged control group. A two-sided alpha level of 0.05 was considered significant. The statistical tests were performed using the GraphPad Prism 9 software.

## 3. Results

### 3.1. Persistence of H9N2 AIV in Feces from Virus-Inoculated Chickens and Spiked Fecal Material

To address the first objective, the current study demonstrated viability of H9N2 AIV in the feces of infected chickens and experimentally spiked fecal material in models A and B. To determine virus viability in model A, infected fecal material was collected from the non-absorbable mats immediately after replacing the seeder chickens with exposed chickens on day 3 PI (0 h PE). In model B, virus titers in the collected fecal material were determined every 12 h starting from 0, 6 h post addition of exposed chickens, until no viable H9N2 AIV titers were detected in the samples. 

**Figure 1:**
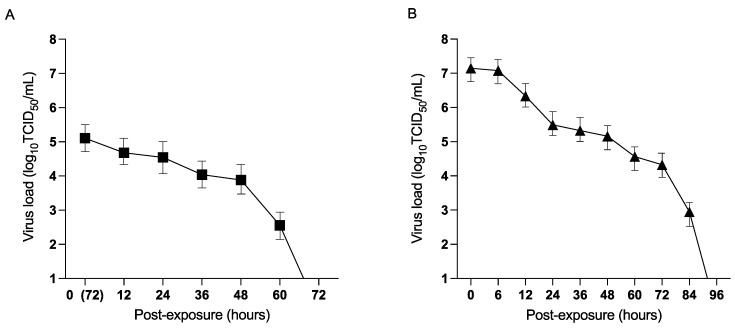
Persistence of H9N2 AIV in feces: (**A**) represents the persistence of H9N2 AIV in feces from infected chickens in model A. On day 14 of age, seeder chickens in model A were inoculated with H9N2 AIV via direct inoculation or PBS (control), respectively (*n* = 10). Seeder chickens were held for 3 days post-infection (PI) and then removed without disturbing the internal settings of the isolator. A group of healthy naïve chickens (*n* = 10) were then exposed to the feces from the seeder chickens. Fecal samples were collected immediately after the addition of the exposed chickens in the isolator (0 h PE). The collected samples were placed in petri dishes within the Horsfall units. Samples were taken from the collected fecal droppings and assessed for virus load every 12 h starting from 0 h PE. (**B**) represents persistence of H9N2 AIV in experimentally spiked fecal droppings in model B. Fecal samples from healthy uninfected chickens (*n* = 10) were collected and experimentally spiked with H9N2 AIV. The inoculum was deposited in different areas of the Horsfall units. Fecal samples were collected from the deposited inoculum immediately after the introduction of the exposed chickens (0 h PE). Virus load (log_10_ transformed) in the experimentally spiked fecal droppings was assessed every 12 h starting from 0, 6 h PE. Virus load (log_10_ transformed) in the fecal droppings in both the models was assessed based on TCID_50_ assay.

The results from this study revealed that H9N2 AIV can persist in chicken fecal material after being disseminated into the surroundings. In model A, H9N2 AIV was present for a shorter duration and remained detectable for up to 60 h PE (Figure 1A). Viral load in model A was sharply reduced from 5.1 log_10_ TCID_50_/mL to 4.6 log_10_ TCID_50_/mL within 12 h PE. This reduction continued, however at a lower rate, reducing from 4.5 log_10_ TCID_50_/mL at 24 h to 2.4 log_10_ TCID_50_/mL at 60 h PE.

In model B, H9N2 AIV remained viable and showed detectable titers up to 84 h PE (2.8 log_10_ TCID_50_/mL) (Figure 1B). The viable H9N2 AIV present in the spiked fecal inoculum was 2 logs higher at 0 h PE, with average virus titers of 7.1 log_10_ TCID_50_/mL PE when compared to 5.1 log_10_ TCID_50_/mL in model A. Virus viability declined five-fold to 5.5 log_10_ TCID_50_/mL between 0–12 h PE. However, there was no substantial decline in the infectious titers between 24 h (5.5 log_10_ TCID_50_/mL) and 48 h (5.1 log_10_ TCID_50_/mL) PE over the timeline. A gradual decline in H9N2 AIV titers was observed between 60 to 84 h PE, with no viable H9N2 AIV titers beyond 84 h PE in the spiked fecal samples.

**Figure 2:**
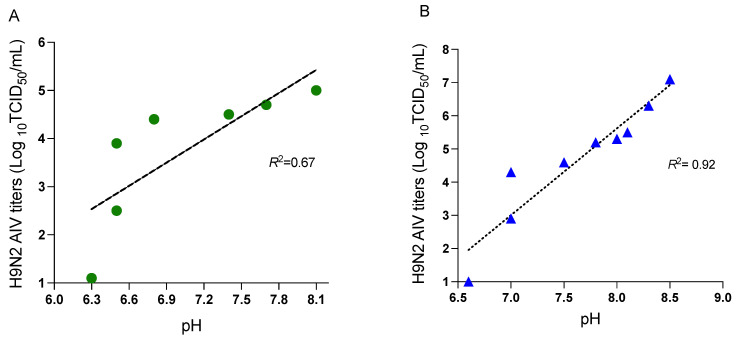
Correlation of H9N2 AIV viability in the context of pH in models A and B: Correlation between H9N2 AIV titers (log_10_ transformed) and pH in fecal droppings from infected birds in model A (**A**) and experimentally spiked fecal droppings in model B (**B**) were determined post-exposure (PE) at different time points using Pearson’s correlation test. Scatter plots illustrate the magnitude of correlation between the H9N2 AIV titers (TCID_50_/mL) and pH at different time points in both the models.

The results further revealed a gradual decline in pH of the fecal samples in both models. The decline in pH was observed to be associated with reduced virus titers at different time points (Figure 2). In model A, pH in the contaminated feces from H9N2 AIV-infected chickens declined from 8.1 to 6.3 between 0 to 60 h PE (Figure 2A). In contrast, in model B, the pH of the spiked fecal droppings was in a range of 8.3 to 7.0 between 0 and 84 h PE and further declined to 6.6 at 96 h, where no detectable H9N2 AIV titers were observed (Figure 2B). Pearson’s correlation test was further used to determine any possible association between pH and virus titers. In model A, higher titers of H9N2 AIV (>5.0 log_10_ TCID_50_/mL) were observed at an average pH ranging between 8.1–7.2. In model B, the titers (>5.0 log_10_ TCID_50_/mL) were detected to be within a pH range between 8.5–7.7. In model A, the lowest H9N2 AIV titers were observed at 60 h PE, with an average pH of 6.3. In model B, the lowest detectable virus titer (2.9 log_10_ TCID_50_/mL) was detected at 84 h PE, when the average pH of the samples was 7.0. Therefore, our results implied that there was a direct correlation between virus titers and pH in both models. The magnitude of correlation (*R*^2^) between pH of the spiked fecal inoculum and viable virus titers in model B (Figure 2B) had higher *R*^2^ values at various time points compared to model A (Figure 2A). 

### 3.2. H9N2 AIV Challenge in the Seeder Chickens in Model A

Swab samples were collected from the seeder chickens PI to determine viral shedding at various time points. The infected chickens remained asymptomatic with no overt signs throughout the trial period. The control (PBS+ unchallenged) chickens remained negative and did not show any virus shedding during the experiment. 

**Figure 3:**
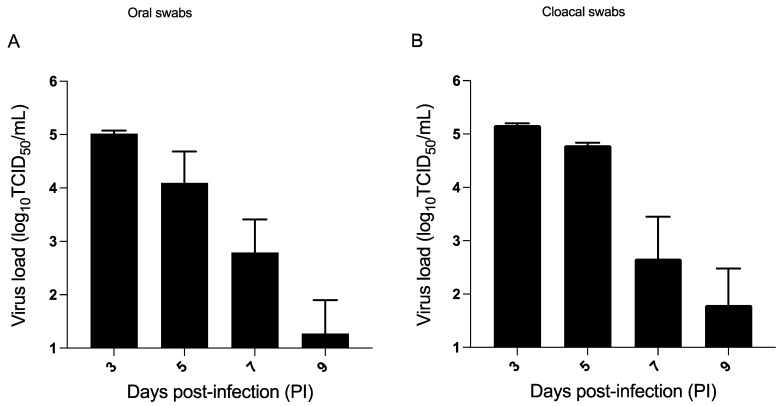
Virus titers in inoculated/seeder group (model A). On day 14 of age, the seeder chickens were infected with H9N2 AIV via direct inoculation route or PBS (unchallenged), respectively (*n* = 10). The PBS unchallenged chickens remained negative and did not show any virus shedding throughout the trial. Virus load (log_10_ transformed) was assessed in oral (**A**) and cloacal swabs (**B**) based on TCID_50_ assay on days 3-, 5-, 7- and 9 PI.

Viral loads were observed in oral (Figure 3A) and cloacal (Figure 3B) swabs on days 3, 5, 7 and 9 PI. The peak in oral shedding was observed on day 3 PI, with an average titer of 5.0 log_10_ TCID_50_/mL, which declined to 1.3 log_10_ TCID_50_/mL on day 9 PI. The chickens did not show any detectable virus shedding beyond day 9 PI (Figure 3A). In terms of cloacal shedding, the seeder chickens exhibited cloacal shedding on days 3, 5, 7 and 9 PI. Similar to oral shedding, maximum AIV shedding in the cloacal swabs occurred on day 3 PI (5.2 log_10_ TCID_50_/mL) (Figure 3B) and declined by day 9 PI. The highest number of chickens positive for H9N2 AIV infection (based on oral and cloacal shedding) was on day 3 PI (10/10), followed by day 5 PI (8/10), day 7 PI (7/10) and day 9 PI (4/10). 

### 3.3. H9N2 AIV Transmission to Exposed Chickens

The results revealed that H9N2 AIV transmission to exposed chickens could occur by contaminated feces from infected chickens as well as from the experimentally spiked feces deposited in different locations within the isolator. The exposed chickens of model B showed an overall higher amount of infection, attributed to the higher oral and cloacal H9N2 AIV load recovered at different time points, compared to the exposed chickens of model A.

**Figure 4:**
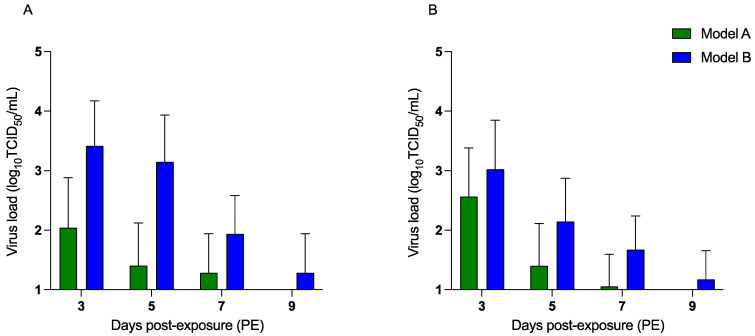
H9N2 AIV shedding in the oral and cloacal swabs on days 3-, 5-, 7- and 9 PE in the exposed chickens. Mean H9N2 AIV titers (TCID_50_/mL) in the oral (**A**) and cloacal (**B**) swabs were determined in the exposed chickens (*n* = 10) post-exposure (PE) in models A and B. In model A, H9N2-inoculated chickens (seeder group) were replaced with healthy exposed chickens on day 3 PI and H9N2 AIV shedding was assessed on days 3-, 5-, 7- and 9 PE. In model B, naïve chickens were added immediately after the dissemination of the H9N2 AIV-spiked fecal inoculum. Virus load (log_10_ transformed) in the exposed chickens was assessed based on TCID_50_ assay at the above-mentioned time points.

Oral shedding was observed by day 3 PE in both groups (models A and B). At all-time points, oral shedding in model B exposed chickens was higher than in model A exposed chickens. The peak oral shedding in model A (Figure 4A) occurred on day 3 PE, with an average titer of 2.0 log_10_ TCID_50_/mL. The shedding declined from 1.4 log_10_ TCID_50_/mL on day 5 to 1.2 log_10_ TCID_50_/mL on day 7 PE. The virus was not detectable beyond day 7 PE in the exposed chickens in model A. On the contrary, in model B, AIV titers remained detectable up to day 9 PE in the oral swabs of the exposed chickens. Oral shedding was observed on day 3 PE, with an average virus load of 3.4 log_10_ TCID_50_/mL. Virus titers declined to 3.1 log_10_ TCID_50_/mL on day 5 PE, followed by day 7 (1.9 log_10_ TCID_50_/mL) and day 9 PE (1.2 log_10_ TCID_50_/mL).

Cloacal shedding from the exposed groups (Figure 4B) demonstrated a similar trend as the highest amount of oral shedding was detected on day 3 PE in both models. A higher amount of cloacal shedding was detected in model B exposed chickens at various time points compared to model A exposed chickens. In model A, the titers showed a gradual decline from day 3 (2.5 log_10_ TCID_50_/mL) and day 5 (1.4 log_10_ TCID_50_/mL) to day 7 PE (1.1 log_10_ TCID_50_/mL). No detectable shedding was observed in model A exposed chickens beyond day 7 PE. On the other hand, cloacal shedding from model B exposed chickens lasted up to day 9 PE (Figure 4B). The average cloacal shedding in model B exposed chickens was higher on day 3 (3.0 log_10_ TCID_50_/mL), day 5 (2.1 log_10_ TCID_50_/mL) and day 7 PE (1.4 log_10_ TCID_50_/mL) compared to that in model A. 

### 3.4. Virus Isolation and HI Antibody Titers

H9N2 AIV detected in the oral swabs of exposed chickens suggested that a greater number of exposed chickens were infected in model B at different time points. H9N2 AIV could be detected in 7/10 exposed chickens in model B on day 3 PE compared to 4/10 in model A (Table 2). Model B exposed chickens showed detectable AIV titers until day 9 PE, with 1/10 chickens exhibiting detectable titers in the oral and cloacal swabs. In the case of model A, a lower number of chickens were detected shedding AIV on days 5 (3/10) and 7 PE (2/10), respectively (Table 2). Additionally, in the cloacal swabs (Table 3), 8/10 exposed chickens in model B exhibited detectable AIV shedding on day 3 PE. The numbers declined from 8/10 to 6/10 on days 5 and 7 PE, respectively. In model A, cloacal shedding was detected in 3/10 chickens on days 3 and 5 and declined to 2/10 by day 7 PE (Table 3). In the PBS+ challenged group, the exposed chickens infected via direct contact transmission of the virus demonstrated oral and cloacal shedding at different time points.

**Figure 5:**
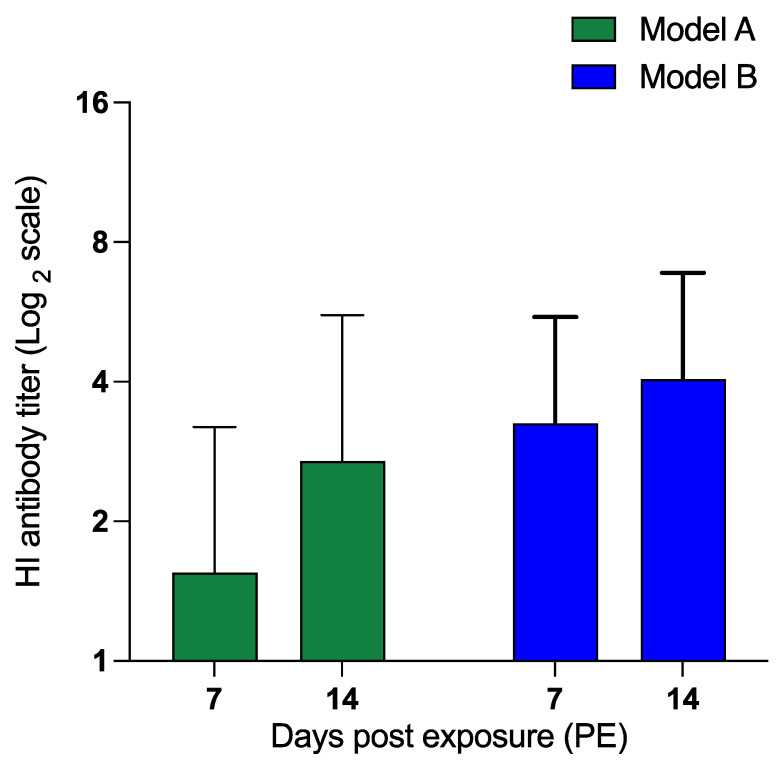
Serum HI antibody titers against H9N2 AIV in exposed chickens. On day fourteen of age, exposed chickens were exposed to contaminated feces from H9N2 AIV-infected chickens (model A) or experimentally spiked feces (model B) or PBS (control) (*n* = 10/group). Serum was collected on days 7 and 14 PE. The HI titers were first observed on day 7 PE. The PBS+ unchallenged chickens remained negative at both time points.

Antibody responses against H9N2 AIV infection were analyzed on days 7 and 14 PE using HI assay to determine differential antibody production and confirm H9N2 AIV infection in exposed chickens. Antibody response was detected in both models, with enhanced titers detected on day 14 PE compared to day 7 PE (Figure 5). Average HI titers in model B exposed chickens were greater on both day 7 (3.2 log_2_ scale) and 14 (4.1 log_2_ scale) PE compared to those in model A exposed chickens on days 7 (1.5 log_2_ scale) and 14 (2.7 log_2_ scale), respectively.

### 3.5. Administration of CpG ODN 2007 and Poly(I:C) Reduces Transmission of H9N2 AIV from Exposed Chickens in the Fecal Transmission Model

**Figure 6:**
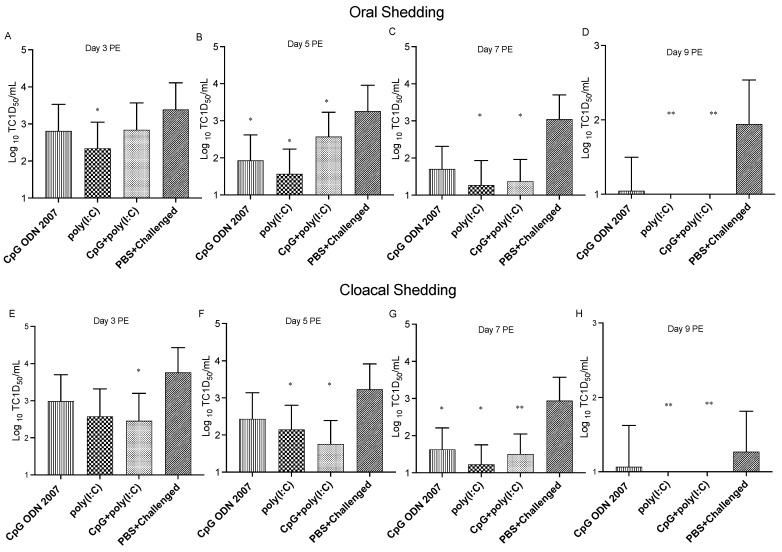
Virus titers in oral and cloacal swabs in the TLR ligand-treated exposed chickens on days 3-, 5-, 7 and 9 post-exposure (PE). The figure represents the mean virus shedding titers (log_10_ transformed) of H9N2 AIV (expressed as TCID_50_/mL) in oral (**A**–**D**) and cloacal swabs (**E**–**H**) on days 3-, 5-, 7- and 9 PE in the exposed groups. The chickens were treated with 100 μL of CpG ODN 2007 (10 μg/chicken) and poly(I:C) (400 μg/chicken), a combination of CpG ODN 2007 (10 μg/chicken) + poly(I:C) (400 μg/chicken) or 100 μL PBS for the positive and negative control group. After eighteen hours of TLR ligand treatment, the treated chickens were exposed to H9N2 AIV-contaminated feces in the isolator (except the negative control group). Statistical analysis was done by one-way ANOVA followed by Tukey’s post hoc test (parametric). When data were non-parametric, a Kruskal–Wallis test was performed. *: *p* < 0.05 or **: *p* < 0.01 (vs. PBS control).

Exposed chickens that were administered poly(I:C) showed a significant decline in oral shedding on days 3 (2.3 log_10_ TCID_50_/mL), 5 (1.6 log_10_ TCID_50_/mL) and 7 PE (1.3 log_10_ TCID_50_/mL) compared to the PBS+ challenged group (*p* < 0.05) (Figure 6A–D). The poly(I:C)- or combination group-treated exposed chickens did not exhibit virus shedding on day 9 PE. The combination-treated exposed chickens had a significant reduction in oral shedding on days 5 (2.6 log_10_ TCID_50_/mL) and 7 PE (1.3 log_10_ TCID_50_/mL) compared to the PBS+ challenged group (*p* < 0.05). Additionally, chickens that received CpG ODN 2007 also showed a significant reduction in oral H9N2 AIV shedding titers on day 7 PE (1.3 log_10_ TCID_50_/mL) (Figure 6C) compared to the PBS+ challenged group (*p* < 0.05). 

**Figure 7:**
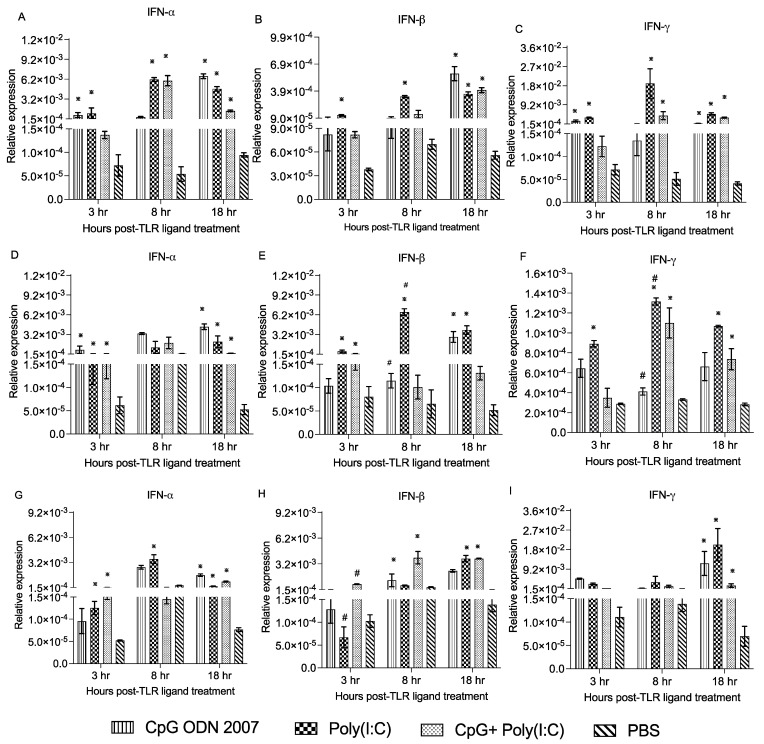
Relative gene expression of IFN-α, IFN-β and IFN-γ in the duodenum (**A**–**C**), jejunum (**D**–**F**) and ileum (**G**–**I**) at 3, 8 and 18 h post-CpG ODN 2007, poly(I:C) and combination treatment. Relative gene expression of IFN-α, IFN-β and IFN-γ at 3, 8 and 18 h post-TLR ligand treatment. Chickens were treated with CpG ODN 2007 (10 μg/chicken), poly(I:C) (400 μg/chicken) and a combination of CpG ODN 2007 (10 μg/chicken) + poly(I:C) (400 ug/chicken), or 100 μL PBS for the negative group. The plotted values represent the mean gene expression levels relative to B-actin ± standard error of the mean (SEM). Statistical significance was calculated using one-way ANOVA followed by Tukey’s multiple comparison test. The results were considered significant from PBS control *p* < 0.05 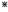 and ^#^: *p* < 0.05 between two treatment groups.

Administration of poly(I:C) also led to a significant reduction in cloacal shedding on days 5, 7 and 9 PE compared to the PBS+ challenged chickens (*p* < 0.05) (Figure 6E–H). Chickens that were administered the combination showed the highest reduction in cloacal shedding compared to the poly(I:C) and CpG ODN 2007 alone groups. There was a significant reduction in cloacal shedding in the combination group-treated chickens on days 3 (2.4 log_10_ TCID_50_/mL), 5 (1.7 log_10_ TCID_50_/mL) and 7 PE (1.5 log_10_ TCID_50_/mL) (Figure 7E–H) compared to the PBS+ challenged group (*p* < 0.05). The CpG ODN 2007-treated chickens showed significant reduction in cloacal shedding on day 7 PE. Moreover, there was no shedding observed from the poly(I:C)- and combination group-treated chickens on day 9 PE (*p* < 0.05).

### 3.6. Treatment with TLR Ligands Induces Antiviral Responses in Different Sections of Small Intestine

To monitor the immune responses, gene expression was analyzed in different parts of the small intestine, i.e., the duodenum, jejunum and ileum. In the duodenum, there was a significant upregulation of interferon-alpha (IFN-α) (Figure 7A) in the poly(I:C)-treated chickens at 3, 8 and 18 h post-TLR ligand treatment compared to the PBS+ unchallenged group. Chickens that received CpG ODN 2007 showed significantly enhanced levels of IFN-α at 3 and 18 h post-TLR ligand treatment compared to the PBS+ unchallenged group. Moreover, IFN-α expression was enhanced at 8 and 18 h post-TLR ligand treatment in the combination group compared to the PBS+ unchallenged group (*p* < 0.05). It was further observed that the poly(I:C) alone treated chicken showed enhanced expression of IFN-ß at 3 and 8 h post-TLR ligand treatment (*p* < 0.05). Chickens treated with CpG ODN 2007, poly(I:C) alone or in combination presented enhanced IFN-ß expression at 18 h post-TLR ligand treatment (Figure 7B) (*p* < 0.05). CpG ODN 2007 alone treated chickens showed significant upregulated expression of interferon gamma (IFN-γ) at 3 and 18 h compared to the PBS+ unchallenged group, whereas treatment with poly(I:C) alone and combination group demonstrated upregulated levels of IFN-γ at 8 and 18 h post-TLR ligand treatment (*p* < 0.05) (Figure 7C). 

**Figure 8:**
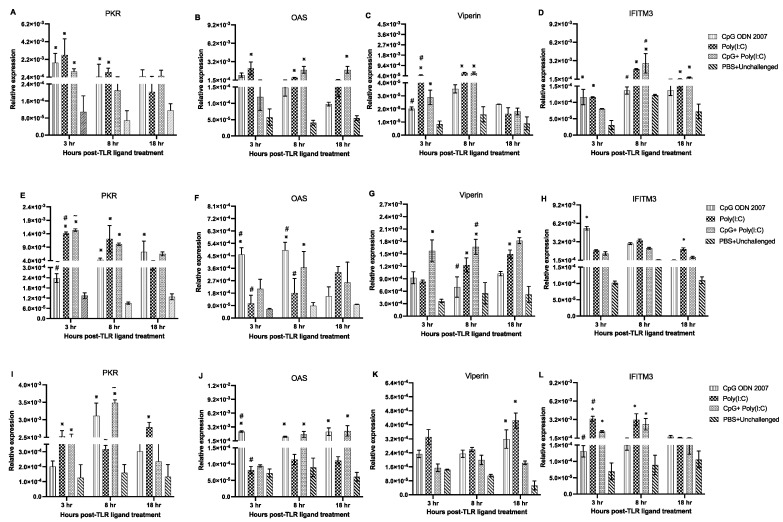
Relative gene expression of ISGs in the duodenum (**A**–**D**), jejunum (**E**–**H**) and ileum (**I**–**L**) at 3, 8, and 18 h post-CpG ODN 2007, poly(I:C) and CpG ODN 2007 + poly(I:C) administration. Relative gene expression of PKR, OAS, viperin and IFITM3 at 3, 8, and 18 h post-TLR treatment. Chickens were treated with CpG ODN 2007 (10 μg/chicken), poly(I:C) (400 μg/chicken) and a combination of CpG ODN 2007 (10 μg/chicken) + poly(I:C) (400 μg/chicken), or 100 μL PBS for the negative group. The values represent the mean gene expression levels relative to B-actin ± standard error of the mean (SEM). Statistical significance was calculated using one-way ANOVA followed by Tukey’s multiple comparison test. The results were considered significant from PBS control *p* < 0.05 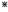 and ^#^/^~^: *p* < 0.05 between two treatment groups.

TLR ligands also induced varied expression profiles of interferon-stimulated genes (ISGs) in the duodenum. Chickens that were administered CpG ODN 2007 and poly(I:C) alone showed an upregulated expression of protein kinase R (PKR) at 3 and 8 h post-TLR ligand treatment compared to the PBS+ unchallenged group (Figure 8A) (*p* < 0.05). Chickens treated with poly(I:C) showed elevated 2′-5′-oligoadenylate synthetase (OAS) expression levels at 3 and 8 h compared to the PBS+ unchallenged group (*p* < 0.05). On the other hand, the combination-treated chickens demonstrated an upregulation in the OAS transcripts at 8 and 18 h post-TLR ligand treatment (*p* < 0.05) (Figure 8B). Chickens treated with poly(I:C) and the combination induced a significant upregulation of viperin at 3 and 8 h compared to the PBS+ unchallenged group (*p* < 0.05) (Figure 8C). Moreover, chickens which received the combination showed upregulated transcripts of interferon-induced transmembrane protein 3 (IFITM3) transcripts at 8 and 18 h compared to the PBS+ unchallenged group (Figure 8D) (*p* < 0.05). poly(I:C)-treated chickens also displayed upregulated IFITM3 expression at 3, 8, and 18 h post-TLR ligand treatment (Figure 8D).

In the jejunum, IFN-α transcripts were upregulated at 3 and 18 h post-TLR ligand treatment in the poly(I:C), CpG ODN 2007 and combination group compared to the PBS+ unchallenged group (*p* < 0.05) (Figure 7D). Chickens that received poly(I:C) exhibited a significant upregulation of IFN-ß transcripts (Figure 7E) at 3, 8 and 18 h post-TLR ligand treatment (*p* < 0.05). A similar response was observed in the poly(I:C)-treated chickens, which showed significant upregulation of IFN-γ expression at 3, 8 and 18 h post-TLR ligand treatment (Figure 7F) (*p* < 0.05). Chickens treated with the combination showed enhanced expression of IFN-γ at 8 and 18 h post-TLR ligand treatment (*p* < 0.05). 

TLR ligands also induced varied expression of ISGs in the jejunum. Chickens that were administered poly(I:C) alone and the combination showed upregulated expression of PKR at 3 and 8 h post-TLR ligand treatment compared to the PBS+ unchallenged group (*p* < 0.05). A significant upregulation of PKR transcripts (Figure 8E) was also observed in the poly(I:C)-treated chickens at 3 h compared to the CpG ODN 2007-treated chickens (*p* < 0.05). With regard to OAS expression, CpG ODN 2007-treated chickens induced significant OAS expression at 3 and 8 h post-TLR ligand treatment (Figure 8F). Chickens that were administered the combination exhibited enhanced expression of OAS at 8 h post-TLR ligand treatment (*p* < 0.05). Chickens that received the combination showed significant expression of viperin (Figure 8G) at 3, 8 and 18 h post-TLR ligand treatment compared to the PBS+ unchallenged group (*p* < 0.05). The poly(I:C)-treated chickens showed upregulation of viperin transcripts at 8 and 18 h post-TLR ligand treatment (*p* < 0.05). Moreover, it was observed that the CpG ODN 2007-treated chickens displayed higher expression of IFITM3 (Figure 8H) at 3 h post-treatment, while the poly(I:C)-treated chickens showed upregulated expression of IFITM3 at 18 h compared to the PBS+ unchallenged group (*p* < 0.05).

In the ileum, the poly(I:C)-treated chickens had higher IFN-α transcripts (Figure 7G) at 3, 8 and 18 h post-TLR ligand treatment compared to the PBS+ unchallenged group (*p* < 0.05). Chickens that received the combination had higher expression of IFN-α at 3 and 18 h post-TLR ligand treatment compared to the PBS+ unchallenged group (*p* < 0.05). The poly(I:C) alone group and the combination group chickens displayed upregulation of IFN-ß expression at 18 h post-TLR ligand treatment compared to the PBS+ unchallenged group (Figure 7H) (*p* < 0.05). Moreover, chickens that received CpG ODN 2007, poly(I:C) alone or in combination demonstrated upregulated IFN-γ transcripts at 18 h post-TLR ligand treatment compared to the PBS+ unchallenged group (Figure 7I) (*p* < 0.05).

Chickens that received poly(I:C) showed upregulated expression of PKR (Figure 8I) at 3 and 18 h post-TLR ligand treatment compared to the PBS+ unchallenged group (*p* < 0.05). The combination group demonstrated a significant upregulation in the PKR transcripts at 3 and 8 h post-TLR ligand treatment (*p* < 0.05). Additionally, OAS transcripts (Figure 8J) were upregulated at 3, 8 and 18 h in the CpG ODN 2007-treated chickens post-TLR ligand treatment compared to the PBS+ unchallenged group (*p* < 0.05). The combination-treated chickens showed significant upregulation of OAS transcripts at 8 and 18 h post-TLR ligand treatment (Figure 8J) (*p* < 0.05). There was an induction of viperin transcripts (Figure 8K) in the CpG ODN 2007- and poly(I:C)-treated chickens at 18 h post-TLR ligand treatment compared to the PBS+ unchallenged group (*p* < 0.05). With respect to IFITM3 levels, chickens treated with poly(I:C) or the combination demonstrated upregulation in IFITM3 expression at 3 and 8 h post-TLR ligand treatment compared to the PBS+ unchallenged group (*p* < 0.05) (Figure 8L).

## 4. Discussion

H9N2 AIV outbreaks have led to severe economic losses in the poultry industry over the past two decades. Fundamentally, the transmission of AIV occurs via close contact with infected individuals or via indirect contact with aerosols or large droplets [9,11,23,40]. However, recent studies have described how transmission of AIV can also occur via contact with contaminated objects (fomites) or feces/slurries [41,42]. The persistence of LPAIVs in feces has been previously demonstrated in wild waterfowl, ducks and poultry [18,19,41,42]. Yet there remains a paucity of information about the transmission of H9N2 AIV via the ‘fecal’ route in chickens. Thus, the present study attempted to establish a fecal model using H9N2 AIV in chickens. Model A involved introduction of naïve chickens to feces from AIV-infected chickens. Model B tested the potential of experimentally spiked fecal droppings deposited in different locations of the Horsfall unit to transmit H9N2 AIV to naïve chickens. The results showed that, as anticipated for an LPAIV, the infected chickens (seeder or exposed) did not show any overt signs throughout the experimental period. 

Our study revealed that the H9N2 AIV could remain detectable in feces from infected chickens for up to 60 h PE and up to 84 h PE in the experimentally spiked feces (model B). AIV titers in the spiked feces were observed to be higher at 0 h PE compared to those in model A. Given the longer duration of H9N2 AIV viability in model B, the higher load of H9N2 AIV at 0 h PE in model B may have impacted the duration of AIV viability in the course of the experiment. This finding aligns with previous studies by Lu and colleagues (2003), which demonstrated that H7N2 LPAIV could remain infective in experimentally prepared chicken manure for up to 35–40 h [17]. Moreover, studies by Thompson and colleagues (2017) found that the viability of AIVs can vary with the virus load present in the surroundings [8].

The difference in the immediate AIV load in feces in both models (0 h PE) could also be attributed to the nature of the experimental approaches used in the present research. In model A, seeder chickens were inoculated individually with H9N2 AIV [24,43]. Hence, the virus load present in feces of model A depended on the overall virus titers in the cloacal shedding post-infection. In model B, the feces were experimentally spiked with a known dose of H9N2 AIV and disseminated among the exposed chickens [12,41]. 

We further determined that H9N2 AIV titers were higher at an alkaline than at a neutral pH. In model B, the spiked feces had a basic to neutral pH at initial time points compared to model A. This could be partly related to the buffering capacities associated with the preparation of spiked fecal inoculum in double-distilled water. Hence, a basic pH in feces at the initial time points may have provided a buffering effect on H9N2 AIV in models A and B. This finding can be supported by previous studies which have reported that a neutral to basic pH of compost and litter material can impart buffering capacities to sustain the viability of infectious AIV particles in organic matter [12,42,44,45,46]. In a recent study, Figueroa and colleagues (2021) highlighted that LPAIVs rapidly become inactivated in acidified broiler litter [41]. The decrease in AIV titers in feces in both models could be related to the acidic pH at later time points. It is suggested that an acidic pH below 6.3 can cause loss of haemagglutinin (HA) glycoprotein activity, leading to irreversible antigenic and conformational changes in the fusion proteins [47]. 

Furthermore, infection in the exposed chickens of both models confirmed that transmission of H9N2 AIV could occur from contaminated feces. This finding can be supported by previous studies which have mentioned that transmission of LPAIV in wild waterfowl can occur via ingestion of contaminated feces, sediments, feed and water present in environmental surroundings [19,48,49,50,51]. Transmission of AIV from feces to exposed chickens can occur via multiple routes. The most common route that has been widely studied is the oral–fecal route of transmission [50]. However, recent reports have outlined other alternative ways of AIV transmission from contaminated feces. For example, phenomena like cloacal drinking and preening reported in ducks and chickens facilitate the uptake of AIVs via contractile movements of the cloaca. ‘Preening’ involves virus uptake due to the dabbing behavior in avian species [52,53]. Generation of aerosolized fomites from fecal material due to the social behavior of chickens may also contribute to overall infection in exposed chickens [10,48,50,53,54].

A higher amount of oral and cloacal shedding was detected in the exposed chickens of model B compared to model A. This could be related with the higher H9N2 AIV load present in the spiked feces in model B. It has been previously shown that the magnitude of AIV infection depends upon the dose of the virus used for infection [55]. In the present study, there was a higher virus shedding via the cloacal route compared to the oral route in both models. This could be attributed to the GIT being the primary site of AIV replication [25,56]. Moreover, it could also be due to virus uptake via a cloacal drinking mechanism, leading to a higher cloacal shedding of AIV [53]. 

Serum antibodies against H9N2 AIV in the exposed chickens of models A and B confirmed the establishment of infection. Model B exposed chickens showed a greater magnitude of antibody-mediated responses against H9N2 AIV. This can be ascribed to the higher amount of infection in the exposed chickens [57,58]. It has been previously demonstrated that the induction of innate responses in cecal tonsils can orchestrate the magnitude of local as well as systemic responses in the GIT of chickens to confer protection against AIV infection [59,60].

We then examined the effects of TLR ligands on the transmission of AIV. The exposed chickens treated with CpG ODN 2007, poly(I:C) alone or in combination showed a decrease in AIV shedding at various time points. The poly(I:C)- and combination-treated chickens demonstrated the highest reduction in AIV shedding, followed by the CpG ODN 2007 group. These results are in alignment with previous studies that indicated that administration of TLR ligands induces innate anti-viral and pro-inflammatory responses that interfere with virus replication and reduce virus shedding [29,61,62,63]. A possible cause for the higher reduction in shedding from the poly(I:C)-treated exposed chickens could be the downstream signaling via two pathways; i.e., the toll-interleukin-1 receptor (TIR) domain-containing adaptor-inducing IFN (TRIF) and interferon regulatory factor (IRF) pathway and melanoma differentiation-associated gene 5 (MDA-5) pathway. Thus, it is plausible that the utilization of these two downstream activation pathways may have had an additive or synergistic response to production of type I and II IFNs. Our present results from the gene expression analysis also support that the poly(I:C)-treated exposed chickens exhibited a higher induction of type I and II IFNs at all time points. Poly(I:C) has previously demonstrated an upregulated expression of type I and II IFNs, showing protective anti-viral responses against AIV infection [29,61,64]. Furthermore, the reduction of oral shedding in the CpG ODN 2007 group can be attributed to the upregulation of type I and II IFNs in the duodenum at various time points, with the highest expression of OAS and PKR in the jejunum following the cecum. This result aligns with previous studies demonstrating the anti-viral role of CpG ODNs in AIV replication in chickens [29,34,65]. The combination-treated chickens exhibited a decrease in oral and cloacal shedding, with the maximum reduction in the cloacal virus titers. This could be related to the upregulation of type I and II IFNs, PKR, OAS and viperin in the jejunum and ileum. The synergistic response could possibly be due to the utilization of different adaptor molecules in downstream pathways [MyD88 by CpG ODN 2007 and TRIF by poly(I:C)] [62], which could have enhanced the expression of IFNs in different segments of the GIT. This is in agreement with previous studies that have highlighted that the co-stimulation of chicken monocytes with CpG ODN and poly(I:C) can upregulate cytokine expression and production of nitric oxide (NO) against viral infections [62,66].

In conclusion, the present study highlighted that feces from H9N2 AIV-infected chickens can act as a source of transmission to naïve exposed chickens. AIVs can survive in feces for a period of time at a neutral to basic pH. The transmission of the virus in the chickens exposed to contaminated feces varies with the differential uptake of the virus by every chicken in the exposed group and the titer of AIV present in the feces. Moreover, we confirmed that employing TLR ligands can be an effective antiviral strategy to prevent AIV transmission from feces to naïve chickens. Future studies should focus on the effect of factors such as temperature and humidity on H9N2 AIV survival in the environment and identify molecular pathways by which TLR ligands enhance immunity in the naïve chickens infected via fecal contact transmission.

## Figures and Tables

**Table 1:** Primer sequences used for quantitative real-time polymerase chain reaction.

Gene	Primer Sequence	Annealing Temperature	References
ß-actin	F:5′-CAACACAGTGCTGTCTGGTGGTA-3′	58	[34]
	R: 5′-ATCGTACTCCTGCTTGCTGATCC-3′		
IFN-γ	F: 5′-ACA CTG ACA AGT CAA AGC CGC ACA-3′	60	[38]
	R: 5′-AGT CGT TCA TCG GGA GCT TGG C-3′		
IFN-α	F: 5′-ATCCTGCTGCTCACGCTCCTTCT-3′	64	[39]
	R: 5′-GGTGTTGCTGGTGTCCAGGATG-3′		
IFN-β	F: 5′-GCCTCCAGCTCCTTCAGAATACG-3′	64	[39]
	R: 5′-CTGGATCTGGTTGAGGAGGCTGT-3′		
PKR	F: 5′-TGGTACAGGCGTTGGTAAGAG-3′	60	[32]
	R: 5′-GAGCACATCCGCAGGTAGAG-3′		
IFITM3	F: 5′-CACACCAGCATCAACATGCC-3′	60	[32]
	R: 5′-CCTACGAAGTCCTTGGCGAT-3′		
Viperin	F: 5′-GGAGGCGGGAATGGAGAAAA-3′	60	[32]
	R: 5′-CAGCTGGCCTACAAATTCGC-3′		
OAS	F: 5′-AGAACTGCAGAAGAACTTTGT-3′	60	[39]
	R: 5′-AGAACTGCAGAAGAACTTTGT-3′		

IFN, interferon; PKR, protein kinase R; IFITM3, interferon-induced transmembrane protein 3; OAS, 2′-5′-oligoadenylate synthetase.

**Table 2:** Virus isolation from oral swabs of exposed chickens in models A and B (*n* = 10).

No. of Swabs Positive/No. of Swabs Tested			
Oral Swabs (Days PE)	Model A	Model B	PBS + Challenged
3	4/10	7/10	9/10
5	3/10	6/10	9/10
7	2/10	5/10	6/10
9	0/10	0/10	5/10

PE, post-exposure.

**Table 3:** Virus isolation from cloacal swabs of exposed chickens in models A and B (*n* = 10).

No. of Swabs Positive/No. of Swabs Tested			
Cloacal Swabs (Days PE)	Model A	Model B	PBS + Challenged
3	3/10	8/10	8/10
5	3/10	6/10	8/10
7	2/10	6/10	7/10
9	0/10	1/10	6/10

PE, post-exposure.
